# Epigenetic and chromatin-based mechanisms in environmental stress adaptation and stress memory in plants

**DOI:** 10.1186/s13059-017-1263-6

**Published:** 2017-06-27

**Authors:** Jörn Lämke, Isabel Bäurle

**Affiliations:** University of Potsdam, Institute for Biochemistry and Biology, Karl-Liebknecht-Strasse 24-25, 14476 Potsdam, Germany

## Abstract

Plants frequently have to weather both biotic and abiotic stressors, and have evolved sophisticated adaptation and defense mechanisms. In recent years, chromatin modifications, nucleosome positioning, and DNA methylation have been recognized as important components in these adaptations. Given their potential epigenetic nature, such modifications may provide a mechanistic basis for a stress memory, enabling plants to respond more efficiently to recurring stress or even to prepare their offspring for potential future assaults. In this review, we discuss both the involvement of chromatin in stress responses and the current evidence on somatic, intergenerational, and transgenerational stress memory.

## Introduction

Climate change is expected to increase the prevalence of extreme environmental conditions, including extreme weather events and increased average temperatures. Crop yield losses that are connected to these changes are inevitable [1, 2]. Thus, improved stress tolerance is a major breeding target. The acute responses to different stresses are relatively well studied, but in nature stress is often chronic or recurring and responses to this type of stress are much less understood. Recent studies suggest that plants have a stress memory that supports adaptation to recurring stress [3–7]. One possible, but largely unexplored, way to improve stress tolerance in crops may thus be to enhance the stress memory through the activation of priming responses or the targeted modification of the epigenome.

The structure of chromatin regulates the accessibility of genes for the transcriptional machinery, and is thus an integral part of regulated gene expression in stress responses and development [8, 9]. In essence, the positioning and spacing of nucleosomes as well as their posttranslational modification, together with methylation of the DNA, affect both the overall packaging and the accessibility of individual regulatory elements. The basic units of chromatin are the nucleosomes, consisting of histone octamers of two molecules each of histone H2A, H2B, H3, and H4, around which 147 bp of DNA are wrapped in almost two turns. The length of thee unpackaged linker-DNA sections between two nucleosomes varies, and this—together with binding of the linker histone H1—contributes to overall packaging. Chromatin structure is further altered by the posttranslational modification of histone tails (e.g., acetylation, methylation, phosphorylation, and ubiquitination), the occupancy and precise positioning of nucleosomes, and the incorporation of histone variants that replace the canonical histones. In addition, the DNA can be modified by cytosine methylation that affects the accessibility of the underlying DNA sequence but does not change the genetic code or base pairing. In plants, cytosines may be methylated in all sequence contexts (CG, CHG, or CHH) and, depending on the context, symmetrical and asymmetrical DNA methylation is distinguished [10, 11]. Symmetrical (CG) DNA methylation has a straightforward mechanism of inheritance through DNA replication; replication results in two hemi-methylated daughter strands and a DNA methyltransferase can be recruited to these sites to fill in the missing methylation mark on the newly replicated daughter strand. Owing to this faithful mode of mitotic inheritance, symmetrical DNA methylation is often referred to as an epigenetic mark (Box 1).

Here, we review the current knowledge on chromatin-based stress memory in the model plant species *Arabidopsis thaliana*. After briefly reviewing the role of chromatin regulators in acute stress responses, we focus on somatic and inherited stress memory. Given the many reviews on priming and stress memory published in the past decade that cover physiological and ecological aspects in model and crop plants [3–7, 12, 13], we focus on cases in which some insight on the molecular mechanism is available. We also discuss emerging general principles. Finally, we consider future directions for research in studying the epigenetics of stress response and their application for crop improvement.

## Box 1. Definition of specific terms used in this review

**Epigenetic phenomenon**—A stable and heritable (through cell divisions) change in gene expression that is independent of DNA sequence changes and is, in principle, reversible.

**Epigenetic modification**—A term commonly used to describe a change in nucleosome structure caused by histone modifications, histone variants, or modification (methylation) of the DNA. These changes are not necessarily epigenetic (see ‘epigenetic phenomenon’) in the sense that they are stable through cell divisions, but (such as symmetrical DNA methylation) some might be.

**Fig. 1:**
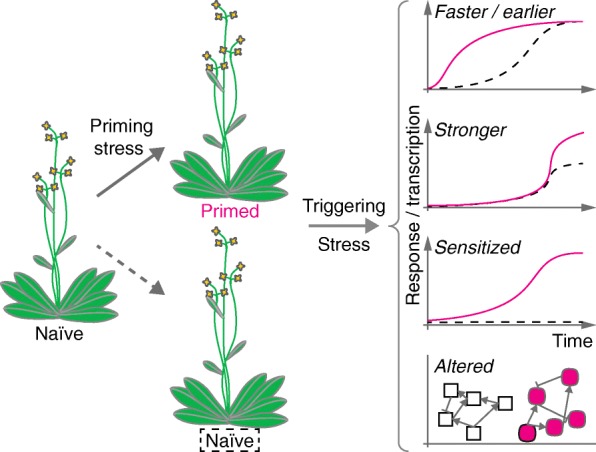
Priming modifies responses to a triggering stress cue. A naϊve plant may be primed by exposure either to stress or to other priming cues such as volatiles. Upon exposure to a triggering stress cue, the response pattern differs markedly in primed and naïve plants. The primed plant may respond to the triggering stress cue faster/earlier or more strongly than a naïve plant. It may also respond in a sensitized fashion so that the response is triggered at a lower threshold. The primed plant may further change its response pattern to regulate a network of genes that differs from that involved in a naïve plant. None of these responses are exclusive and combinations thereof probably occur

**Priming**—Phenomenon through which a transient biotic or abiotic stress cue leads to modified (typically faster or stronger) defense responses upon exposure to a recurring stress (cf. Fig. 1). Described for immunity and for responses to various abiotic stresses.

**Stress memory**—Describes the phenomenon through which information on a past stress cue is retained and results in a modified response upon a recurring stress or a sustained response after the priming stress cue (see ‘priming’).

**Transcriptional memory**—Sustained differential response in gene expression after an exogenous cue. Transcriptional memory can be evident from either sustained changes in expression (activation or repression) or from a modified response after a second cue.

**Memory genes**—Genes that show transcriptional memory.

**Somatic stress memory**—Stress memory whose duration is limited to one generation of organisms. It may be mitotically heritable, but often lasts only a fraction of the lifespan of the organism.

**Transgenerational and intergenerational stress memory**—A stress imprint that extends from one stressed generation of organisms to at least the first stress-free offspring generation. In this review, we use the term ‘intergenerational memory’ when only the first stress-free generation has a detectable memory effect, and ‘transgenerational memory’ if memory is detectable after at least two stress-free generations. As the progeny develops on the mother plant, intergenerational memory may be mediated by the conditions in which the seed grows and by cues introduced into the seed or embryo by the mother plant. Transgenerational memory, by contrast, probably has an epigenetic basis.

## Priming and stress memory

Stress in plants is caused by extreme growth conditions that inhibit normal growth and development and which may be lethal in extreme cases. Such conditions may be caused, for example, by extreme temperatures, too little or too much water (drought or flooding, respectively), or pathogen and herbivore attack. Priming of organismal responses to stress describes the phenomenon by which a temporally limited environmental stimulus (a ‘priming stress cue’) modifies a plant for future stress exposure (a ‘triggering stress cue’) [5, 6]. The term priming was originally coined in the context of immunity against pathogens (biotic stress), but was later also applied to responses to abiotic environmental conditions. While in the primed state, the plant responds to the triggering stress cue with a response that is modified when compared to that of a plant in the naïve (unprimed) state (Fig. 1). Priming acts at the phenotypic level and does not introduce changes in DNA sequence and is thus reversible eventually [5, 6]. Generally, such priming is evidenced by a stronger or faster response pattern, as can be exemplified by the modified activation kinetics of defense gene expression.

The priming event is directly followed by a period of stress memory [14]. This involves the storage of information on the priming stress cue after the cessation of the stress, and can be detected as a modified response to the triggering stress cue when compared to that of a naïve plant. The duration of this memory may often be in the range of days to weeks for somatic stress memory, but in some cases may extend to the offspring (inter- or transgenerational stress memory). Similarly, a memory of an exogenous cue may also occur in stress-independent contexts (for example, during vernalization; see below). One possible manifestation of the memory is a modified transcriptional response (transcriptional memory) [15, 16], during which the priming stimulus induces either sustained changes in gene expression (activation or repression) or a modified transcriptional response (such as hyperinduction) upon a secondary stimulus. Other mechanisms also exist and may involve transcriptional feedback loops (such as autoactivation of a transcription factor) or posttranslational mechanisms (influencing protein stability or protein modifications) [17]. Another form of a self-perpetuating memory that is independent of transcription is the transmission of prions or prion-like proteins, whose mutant conformation induces the conversion of wild-type proteins into the prion state [18–20]. Notably, prion switching in response to environmental stress has been described in yeast [21]. Also in yeast, transcriptional memory of galactose-induced transcription is transmitted cytoplasmically and depends on the galactokinase Gal1 [22–24]. Chromatin-based processes in the nucleus—such as SWI/SNF-dependent chromatin-remodeling, the inclusion of histone modifications and variants, and subnuclear localization—are also involved in this and other examples of transcriptional memory in yeast [23–25].

For every case of stress memory, the possibility of an epigenetic basis must be confirmed. By definition, this requires that the phenomenon is both stable and heritable (through cell divisions), yet independent of DNA sequence change and thus at least in principle reversible. A truly transgenerational stress memory is very likely to be epigenetic, but this may not hold for somatic stress memory because of the shorter duration. It is not yet clear whether many of the observations that we review here can indeed be labeled ‘epigenetic’ in the strict sense of the definition (Box 1). In addition, the term ‘epigenetic mechanisms’ has been adopted by the scientific literature to encompass all of the parameters that impact on the structure of chromatin, including DNA methylation, whether or not they are stably inheritable. This term provides a convenient label for chromatin modifications (both on histones and DNA) and thus is hard to eradicate, but this wide definition has caused considerable confusion. Consequently, in the scientific field, the view has gained acceptance that the term ‘epigenetic mechanisms’ should only be used when referring to truly epigenetic phenomena.

## The role of chromatin in acute stress responses

Chromatin has long been viewed as the interface between the environment and the genome. The flexibility and dynamics of chromatin influence the accessibility of gene loci to the transcription machinery and hence modulate the interpretation of the information encoded in the DNA sequence (reviewed in [26–28]). To illustrate the intricate connection between stress responses and chromatin regulation, we highlight a few recent examples here. Many more studies have reported a link between chromatin-based mechanisms and stress-responsive gene expression, and we refer to several recent reviews that cover different aspects of the subject [7, 12, 29, 30].

Stress-induced transcription factors may directly recruit histone-modifying complexes. A pertinent example of this is the specific recruitment of the COMPASS H3K4 methyltransferase complex by stress-activated bZIP transcription factors [31]. The histone lysine methyltransferases SDG8 and SDG25 have been shown to regulate plant immunity through H3K4 and H3K36 methylation of defense-related target genes [32]; how they are targeted to specific loci, however, remains unclear. The remodeling of nucleosomes is another stress-related chromatin modification that plays an important role in abscisic acid (ABA)-mediated stress responses. There is now convincing evidence that the SWI/SNF chromatin remodeling protein BRAHMA (BRM) represses ABA target genes in the absence of stress. BRM has emerged as a direct target of the ABA signaling cascade and its activity is regulated by ABA-dependent phosphorylation [33, 34]. Other chromatin remodelers of the same class have been implicated in defense responses and growth arrest in response to environmental perturbations [35–37]. Furthermore, DNA methylation and demethylation pathways play a role in the adaptation to non-viral pathogens [30], although the exact mechanisms involved in these defense responses remain elusive.

At the experimental level, it is often challenging to distinguish correlation and causality. Moreover, the fact that a chromatin regulator is required for a certain stress response does not necessarily mean that it actively controls that stress response [12]. It may simply be involved in bringing about changes in gene expression that come with this response rather than a participant in the endogenous regulation of the process. Moreover, the knockout of a chromatin regulator may produce altered stress responses not because of direct or specific regulation of stress responsive genes, but rather as an indirect consequence of developmental, morphological, or metabolic alterations.

## Somatic stress memory

Most responses to abiotic stress exposure or pathogen attack that involve chromatin features are transient and return quickly to baseline levels after normal conditions have been restored. In some cases, however, a more sustained response and evidence of a somatic stress memory have been observed (Box 1 and Table 1) [3, 5, 6, 38]. Vernalization provides a classic example of environmentally mediated epigenetic gene silencing. Vernalization is the acceleration of the transition to flowering by a prolonged period of cold temperatures (usually winter). The vernalization memory is stored for weeks to months after the cold has subsided [39]. In *Arabidopsis thaliana*, this involves epigenetic silencing of the *FLOWERING LOCUS C* (*FLC*) gene through H3K27 trimethylation [40–42]. This histone modification is deposited at the *FLC* locus by a cold-activated polycomb group complex that is targeted through *cis* elements and non-coding RNAs. Although cold temperature is the trigger of this epigenetic switch, it is considered to be a developmental signal.

**Table 1:** Examples of somatic and transgenerational stress memory

Stress cue	Maximal duration of memory (as analyzed)	Plant-level effect	Chromatin marks associated with priming	Protein regulators	Reference(s)
Somatic stress memory					
Desiccation	5–7 d	Yes	H3K4me3, paused RNA Pol II		[63]
Desiccation	4 d	ND	H3K4me3		[64]
Hyperosmotic	10 d	Yes	H3K27me3		[57]
Salt	5 d	Yes	H3K4me3	HY5	[71]
Heat, cold, or salt	7d	Yes	H3K14ac, H3K4me2, H3K4me3	HAC1	[70]
Heat	3 d	Yes	H3K4me2, H3K4me3	HSFA2	[54]
Heat	3 d	Yes	Histone occupancy	FGT1	[58]
Systemic acquired resistance	4–6 d	Yes	H3K4me2, H3K4me3	HSFB1	[43, 46]
Defense priming	ND	Yes	Histone occupancy, H3K4me3	CAF-1	[61]
Inter-/transgenerational stress memory					
Hyperosmotic	Inter-generational	Yes	DNA methylation	DNA methylation, DNA demethylation	[95]
Iron deficiency	Inter-generational	Yes			[93]
Various	Inter-generational	ND			[81]
Bacterial infection, chemical stressors	Inter-generational	Yes	DNA methylation		[99]
Bacterial infection	Trans-generational	Yes	H3K27me3, DNA methylation	DNA methylation	[98]
Caterpillar herbivory	Trans-generational	Yes	DNA methylation	NRPD2A, NRPD2B, DCL2/DCL3/DCL4	[100]

*d* days, *ND* not determined

### Role of histone methylation

The involvement of chromatin modifications in stress priming was first reported in systemic acquired resistance (SAR) after changes were observed after treatment with bacterial pathogens or with the salicylic acid (SA)-analogon acibenzolar S-methyl, a benzothiadiazole (BTH) [43]. This priming was associated with sustained changes in histone modifications at several loci that showed priming-dependent transcriptional memory after a lag phase of several days. In particular, histone H3K4me2 and H3K4me3 were enriched in primed leaves. In line with the systemic nature of SAR, the changes in histone modifications were also found in leaves that were not treated with the priming stress cue. Throughout the genome, H3K4me3 correlates well with gene expression, but the association of H3K4me2 with active transcription is less pronounced [44]. Both modifications have also been implicated in other stress memory phenomena and they may mark chromatin that is poised for transcription more generally [45]. Priming of defense-related genes is lost in mutants in which the transcriptional regulator *HEAT SHOCK FACTOR B1* (*HSFB1*) is lost [46].

Heat stress is highly fluctuating in nature. Sublethal heat stress primes a plant to withstand subsequent high temperatures that are lethal to an unadapted individual. The acute responses to heat are generally referred to as heat shock response and the core mechanism is highly conserved across all eukaryotes [47–50]. Recently, it has been realized that plants also have a heat stress memory, during which acquired thermotolerance is actively maintained and which was described first at the physiological level [51–53]. This heat stress memory involves both types of transcriptional memory, sustained induction and enhanced re-induction [53, 54]. A subclass of heat-inducible genes show sustained activation after a priming heat stress and/or enhanced induction upon recurring heat stress. These genes are referred to as memory genes. The transcriptional memory was associated with hypermethylation of H3K4me2 and H3K4me3 that was maintained for at least 2 days after the end of a heat stress [54]. Because not all heat-inducible genes are memory genes, it was possible to show that the observed H3K4 hypermethylation is not a direct consequence of heat-induced transcription, as the heat-inducible *HSP70* gene did not accumulate H3K4 hypermethylation [54]. Similarly, genetic analysis revealed that the activation of memory genes itself did not induce H3K4 hypermethylation; in a mutant in which the transcription factor HSFA2 is defective, memory genes were initially activated but then quickly switched off and H3K4 hypermethylation was reduced. Interestingly, HSFA2 seems to act in a hit-and-run mode, with a peak binding to target genes early after heat shock and a steep decline within a few hours [54–56]. HSFA2 is required, however, for sustained accumulation of H3K4me2/3, which is maintained at high levels for several days [54]. How HSFA2 mediates this sustained chromatin modification remains to be investigated.

To identify chromatin changes after salt priming globally, Sani et al. [57] performed genome-wide profiling of H3K27me3 after a triggering salt treatment on the roots of plants that had been primed with a mild salt treatment 10 days earlier. The dose of the priming salt treatment did not affect morphology, but at the physiological level, it resulted in reduced salt uptake and enhanced drought tolerance upon a triggering stress cue administered after a memory phase of 10 days. The most striking changes at the chromatin level were a decrease in H3K27me3 at the edges of H3K27me3-enriched islands in the genome, resulting in a shortening and fractionation of these islands [57]. In addition, some genes showed a modified upregulation upon a second salt treatment, but no direct correlation with altered histone modifications in these genes could be found.

### Nucleosome occupancy and transcriptional memory

Besides H3K4 hypermethylation, nucleosome remodeling has very recently been implicated in heat stress memory [58]. The *FORGETTER1* (*FGT1*) gene was identified from a forward mutagenesis screen for factors required for the sustained induction of a heat stress memory gene. *FGT1* encodes a putative helicase and interacts with chromatin remodeling complexes including the SWI/SNF chromatin remodeler BRM. Like FGT1, BRM is specifically required for heat stress memory but not for the immediate heat shock response [58]. The sustained induction of memory genes was associated with a sustained decrease in nucleosome occupancy that required the heat-responsive binding of FGT1 to the transcriptional start site of the memory locus. FGT1 is a highly conserved protein that is also present in metazoans, suggesting that it plays additional roles beyond heat stress memory [59, 60]. The questions of how FGT1 interacts with H3K4 hypermethylation and whether it also plays a role in other stress memory phenomena remain to be investigated.

Nucleosome occupancy was also reported to be involved in priming stimulated by chemical agents. The *fasciata 2* mutant, which is defective in the CHROMATIN ASSEMBLY FACTOR-1 (CAF-1) histone assembly complex, shows a molecular phenotype comparable to a constitutive priming response. This is associated with low nucleosome occupancy and high H3K4me3 at primed genes [61]. Under non-sterile growth conditions, the molecular priming is correlated with spurious activation of primed genes. The observed reduced nucleosome occupancy is in agreement with the role of the evolutionarily conserved CAF-1 complex as a histone chaperone depositing histone H3/H4 tetramers onto newly synthesized DNA during DNA replication [62].

Transcriptional memory was also reported in response to desiccation stress treatment in *A. thaliana* [63–65]. Starting from transcriptome profiling, the authors identified genes that responded differently to a repeated desiccation stress than to the first desiccation stress. Besides genes that showed a classic transcriptional memory (enhanced re-induction upon recurring stress), other classes of genes were identified that showed modified induction upon recurring desiccation—some showed enhanced repression, some loss of induction, and others loss of repression [65]. The enhanced re-induction class (e.g., *RD29B* and *RAB18*) has been characterized in most detail [63], and this response has been found to result from higher transcription correlated with sustained H3K4me3 hypermethylation during the recovery period [63, 66]. Moreover, paused transcription elongation was identified as a probable mechanism; this might result from the observed accumulation of RNA polymerase II that was hyperphosphorylated in the serine 5 residue of the C-terminal domain during recovery. The transcriptional memory and the associated chromatin and RNA polymerase II modifications lasted for 5 to 7 days. A mutant in the *ATX1* H3K4 methyltransferase gene [67, 68] was defective in the amplitude of induction but not the primability [63]. As this mutant retains residual H3K4 methylation at the memory genes, the interpretation of this result is complicated. ABA signaling is necessary but not sufficient for *RD29B* transcriptional memory [69]. The mapping of *cis* elements that are required for this memory identified two adjacent ABA-response elements (ABREs). ABREs are bound by ABA-response element binding factors (ABFs). Interestingly, an *abf2 abf3 abf4* triple mutant displayed compromised induction of the memory genes but not loss of memory, suggesting that transcriptional induction and memory are separable at the level of *trans* factors [63, 69]. Thus, an additional factor yet to be identified is required for the memory. Interestingly, ABA treatment is sufficient as a priming stress cue, but as a triggering stress cue, it is not sufficient to induce the transcriptional memory response.

### Priming effectiveness across different stressors

Plants are exposed to various environmental stressors in their natural environment, and priming by one type of stressor can sometimes enhance plant responses to other types of stress (cross-priming). For example, enhanced immunity to bacterial pathogens can be induced by repeated moderate high or low temperature treatments or by moderate salt treatments [70]. This cross-priming correlates with the hyperinduction of pattern-triggered immunity marker genes (*WRKY53*, *FRK1*, and *NHL10*) after a triggering stress cue, as well as with increased accumulation of H3K14ac, H3K4me2, and H3K4me3, and it requires the histone acetyltransferase HAC1. Interestingly, prolonged stress treatment is not efficient as a priming stimulus, indicating that the plant is able to distinguish between repeated short and chronic stress exposure. This cross-priming was effective for 7 days [70].

A crosstalk between different environmental stimuli was also reported in salt-induced somatic stress memory [71]. The *P5CS1* gene shows transcriptional memory (enhanced re-induction) in response to salt stress, and this was associated with high H3K4me3. The same gene was previously identified as a dehydration stress memory gene [64, 65]. Promoter analysis revealed that separate *cis* elements are required for salt and dehydration stress memory [71]. Interestingly, this salt-dependent memory requires light signaling through the ELONGATED HYPOCOTYL 5 (HY5) transcription factor, which binds the promoter within the salt-memory element [71]; this suggests a higher order integration of different environmental signals.

Besides the regulation at the level of nucleosome positioning and modification, chromatin-independent pathways also contribute to heat stress memory, involving diverse microRNA- and protein stability-based mechanisms [53, 72–74]. For example, isoforms of the microRNA *miR156* are induced after heat stress and repression of their target genes is required for the sustained induction of memory genes and for physiological heat stress memory [53]. As *miR156* is an important regulator of developmental transitions, this signaling module may be used to integrate stress memory and plant development.

### Mechanisms of somatic stress memory

**Fig. 2:**
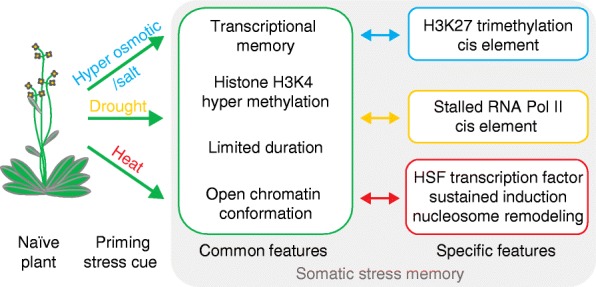
Molecular features of somatic stress memory in response to abiotic stress cues. Somatic priming of plants by an abiotic (hyperosmotic, drought, or heat) stress cue has common features that are displayed in the *central box*. Other properties have as yet only been implicated in a specific stress. Notably, this apparent specificity is based on current knowledge rather than on explicit exclusion (*right boxes*, color-coding as indicated on the *left*). *HSF* heat shock factor

In summary, histone H3K4 methylation is frequently correlated with different types of somatic stress memory (Fig. 2). Interestingly, such a function may be conserved in yeast and metazoans as a mark for transcriptional memory [25, 75]. In addition, reduced nucleosome occupancy during priming has been found in several cases and may be another factor that regulates chromatin-based stress memory [58, 61]. How both mechanisms interact remains to be investigated.

Somatic stress memory is transient and its duration has been studied using enhanced re-induction of gene expression as a read-out. Across different types of abiotic stress priming, the duration was found to be surprisingly similar and lasted 5 to 7 days [63, 70, 71]. The mechanism that limits this duration is currently unknown but may be an interesting target for extending this memory. Duration limits may be connected with the fact that maintaining the primed state requires the allocation of resources (although fewer than would be required to maintain a full defense response). With increasing duration, a point is reached beyond which maintaining the primed state is more costly than new adaptation, and thus resetting becomes advantageous. Moreover, the likelihood that the stress will reoccur decreases with time because many stresses (such as heat waves or attacks by herbivores) occur in clustered patterns. Maintaining a primed state is assumed to be less costly than maintaining the full defense response, not least because full responses often interfere with growth, but few studies have sought to test this hypothesis. One pioneering study on disease priming found that priming does indeed provide a fitness advantage in a situation where disease occurs, whereas it is slightly disadvantageous in a disease-free environment [76]. Studies aiming to determine whether other types of somatic stress memory provide a fitness advantage under field conditions will soon become possible because specific regulators of these processes that do not have pleiotropic effects on growth and development are being identified.

## Transgenerational inheritance of stress memory

Epialleles that are stable for hundreds of years have been identified in plants. The *peloria* mutant of *Linaria vulgaris*, which was identified by Linneus, provides a particularly striking example. The flower of this mutant is radially symmetric (whereas the wild-type flower is dorsoventrally symmetric) because of a methylation change in the promoter of a flower morphogenesis gene [77]. The occasional recovery of revertants that have the wild-type flower phenotype confirms that the phenotype is independent of a DNA sequence mutation. Whether similar epialleles are generated as an adaptation to stress is a subject of intensive study and active discussion [78–80]. To clarify the interpretation of the reported results on potential transgenerational memory, it has been instrumental to assemble a catalogue of criteria and experimental design principles that need to be applied in order to demonstrate transgenerational inheritance [78, 81]. According to this definition, transgenerational epigenetic stress memory is meiotically stable and extends for at least one stress-free generation. Importantly, it is independent of changes in DNA sequence. Nevertheless, because the immediate progeny generation develops on the mother plant, maternal (or even paternal) effects must be taken into account. Thus, we propose to distinguish transgenerational memory (which is detectable until at least the second stress-free generation) from intergenerational stress memory (detectable in the first stress-free generation; Box 1). Transgenerational memory probably has an epigenetic basis, whereas this may or may not be the case for intergenerational memory.

### Mechanisms of stress memory inheritance

Chromatin-based mechanisms of inheritance may involve heritable epialleles that have differential DNA methylation [82–85]. An alternative possibility is that histone modifications are inherited through either nucleosome recycling or the copying of modifications onto newly incorporated histones. The extent to which both processes take place is still under study [86]. Microscopic studies with tagged histones suggest an almost complete replacement of H3 during gametogenesis and fertilization [87]. Nevertheless, this leaves the possibility that a few parental histones are retained or that modifications of the parental histones are copied onto the newly incorporated histones [88]. Interestingly, resetting the vernalized state of *FLC* during embryogenesis requires the activity of an H3K27 demethylase [89]. Mutants lacking the demethylase pass on the vernalized state to their offspring, suggesting that histone-based epigenetic states can, in principle, be transmitted through meiosis and gametogenesis. In yeast, H3K9me at a transgene locus was stable over many mitotic generations in the absence of the EPE1 demethylase [90, 91].

One of the first reports to suggest the existence of a stress-induced transgenerational memory used a somatic homologous recombination (SHR) reporter and demonstrated that SHR increased in the parental generation in response to UV-C irradiation or treatment with the flg22 elicitor [92]. The rate of SHR remained elevated during several unstressed generations, suggesting an epigenetic basis. However, subsequent studies found that effects were observed reliably only in the direct offspring of stressed plants [81, 93, 94], confirming the presence of an intergenerational stress memory.

A very recent study on hyperosmotic stress priming confirmed intergenerational stress memory in the progeny of plants that were stressed during their vegetative development for at least two successive generations [95]. This memory was, however, reset after one stress-free generation, indicating that this is an environmental adaptation that is rapidly lost in the absence of stress [95]. In line with other studies, this intergenerational stress memory appears to be inherited predominantly through the mother. Wibowo et al. elegantly show that paternal inheritance is inhibited by the activity of the DNA glycosylase DEMETER (DME) in the male gametes, and that the transmission of the memory through the father is restored in *dme* mutants [95, 96]. RNA-mediated DNA methylation and DNA-demethylation pathways mediate the intergenerational memory in response to hyperosmotic stress [95]. Indeed, discrete differentially methylated regions (DMRs) that are associated with this memory were identified by genome-wide methylation analysis. These regions are rich in transposable element (TE)-related sequences. Two such DMRs in the promoter of stress-related genes were associated with a priming effect on gene expression that was dependent on intact DNA methylation pathways [95].

Intergenerational or transgenerational stress memory has also been found in response to biotic stresses [13, 97]. Priming in response to *Pseudomonas syringae* was shown to persist into at least the progeny generation [98, 99]. One of the two studies found that the priming persisted into the second stress-free generation, whereas the other did not; notably, slightly different priming stimuli and assay protocols were used. Intergenerational or transgenerational priming was evidenced by enhanced salicylic acid-related defense gene induction and resistance to biotrophic pathogens [98] and by enhanced primability [99]. Mutants that are impaired in DNA methylation pathways showed constitutive priming but an attenuated priming response, suggesting that hypomethylated genes are involved in the priming response [98].

Transgenerational priming was also observed after caterpillar herbivory, a biotic stress that could be substituted for by methyl jasmonate or mechanical damage [100]. Progeny plants showed increased resistance (assayed by decreased caterpillar growth) and increased priming of jasmonic-acid-dependent defense responses. The priming was evident in the second stress-free generation and required jasmonic acid perception and intact small interfering RNA (siRNA) pathways. Thus, both salicylic-acid- and jasmonic-acid-dependent defense responses are primed, and this priming extends at least into the direct progeny generation. This opens up the possibility that this principle could be applied in an agronomic context by priming the parental plants in order to produce more disease-resistant seeds.

Our mechanistic understanding of intergenerational or transgenerational stress memory remains fragmented. Genetic analysis suggests the involvement of DNA methylation and siRNA pathways in several cases. Nevertheless, a contribution from other mechanisms, such as a memory in form of metabolites or proteins deposited in the seed or embryo, remains a possibility, especially where the memory is reset after one stress-free generation. For example, the possible role of phytohormone levels in seeds has been tested in some cases, but has not yet been substantiated [95, 99, 100].

## Conclusions and future directions

The hypothesis that traits that are acquired in one generation could be transmitted to following generations was first put forward by Lamarck in the 19^th^ century. In the early 20^th^ century, this incited Lysenko in socialist Russia to attempt to grow wheat in unsuitable climates with devastating effects. Subsequently, the proposed inheritance of acquired traits was viewed with a sound measure of skepticism, until its popularity was revitalized a few years ago by progress in the field of genomics and epigenetics.

Here, we have reviewed mechanistic insights provided by studies of the annual plant *A. thaliana*. One interesting question is how these insights relate to stress memory in perennials. In perennials, the vernalized state is reset every year after flowering to restore the vernalization requirement for the next growing season [101, 102], but as far as we know, stress memory has not been studied in perennials that are close relatives of *A. thaliana* such as *Arabis alpina*. Trees have an even more extreme life strategy in which an individual may live hundreds or even thousands of years. Using cuttings of hybrid poplar from different environments that were transplanted into a common garden, it has been shown that clone history affects the response to drought in poplar [103]. However, whether this variation in stress response involves gene-targeted chromatin mechanisms has not been studied.

Besides numerous cases of somatic stress memory, a number of well-documented cases of intergenerational stress memory have been identified. From these, a picture emerges in which plants prime their direct offspring for a stress that they may encounter during their lifetime. Given the short life cycle of rapid-cycling *A. thaliana* accessions with more than one generation per year, this may have an obvious adaptive value. In most cases, the memory is reset after one stress-free generation. Correspondingly, it has been suggested that transgenerational inheritance of priming over multiple generations may be disadvantageous in the highly fluctuating conditions of a typical *A. thaliana* habitat [98, 104]. Stress memory in plants with other life strategies remains to be investigated. Similarly, insights into the molecular conservation of stress memory in crop species are scarce. It should be mentioned, however, that the chemical priming of seeds to enhance the stress tolerance and pathogen resistance of young plants after germination is a long-standing agronomic practice, referred to as seed priming. A memory of heat stress has been demonstrated in temperate rice varieties and factors similar to those operating in *A. thaliana* have been implicated [74]. It remains to be investigated whether the priming mechanism is generally conserved in crop species.

Much evidence points towards a prominent role of chromatin-based mechanisms in somatic and intergenerational stress memory, but this does not exclude the involvement of other mechanisms. Progress will undoubtedly be made in unraveling the molecular basis of such stress memory in the coming years. In particular, it will be interesting to see whether different cases of stress memory are encoded by the same mechanisms and whether there are universal stress memory regulators. A major topic will be the need to move beyond correlation by demonstrating that targeted, gene-specific modifications to the epigenome do indeed lead to the anticipated responses. In turn, this will identify key regulatory mechanisms that will allow tailored responses to the challenges represented by the effects of climate change.

Understanding the underlying mechanisms in *A. thaliana* will ultimately enable us to improve stress tolerance in crop species. For example, one possibility might be to exploit stress priming mechanisms to induce a constitutively primed state, thereby increasing a crop’s ability to tolerate stress and disease without at the same time incurring a penalty on biomass accumulation and yield.

## Abbreviations

ABA: Abscisic acid
ABF: ABA-response element binding factor
ABRE: ABA-response element
BRM: BRAHMA
CAF-1: CHROMATIN ASSEMBLY FACTOR-1
DME: DEMETER
DMR: Differentially methylated region
*FGT1*: *FORGETTER1*
*FLC*: *FLOWERING LOCUS C*
SAR: Systemic acquired resistance
SHR: Somatic homologous recombination
siRNA: Small interfering RNA
